# Response to apatinib in chemotherapy-failed advanced spindle cell breast carcinoma

**DOI:** 10.18632/oncotarget.12568

**Published:** 2016-10-11

**Authors:** Na Zhou, Congmin Liu, Helei Hou, Chuantao Zhang, Dong Liu, Guanqun Wang, Kewei Liu, Jingjuan Zhu, Hongying Lv, Tianjun Li, Xiaochun Zhang

**Affiliations:** ^1^ Department of Oncology, The Affiliated Hospital of Qingdao University, Qingdao, China; ^2^ Department of Oncology, Qingdao Municipal Hospital, Qingdao, China; ^3^ Department of Pathology, The Affiliated Hospital of Qingdao University, Qingdao, China

**Keywords:** spindle cell carcinoma, Apatinib, VEGFR, targeted therapy

## Abstract

Spindle cell carcinoma of the breast is a rare subtype of metaplastic carcinoma, and no effective chemotherapy special for metaplastic carcinoma exists until now. As spindle cell carcinomas of the breast are typically “Triple Negative”, endocrine therapy and molecular therapy targeted to Her2 might not be favorable, resulting in poor prognosis. Apatinib is currently being tested in patients with breast or lung cancers. Here we report a successful case using Apatinib to treat spindle cell carcinoma of breast.

A 52- year- old woman presented with a gradually enlarged lump in left breast, which was revealed to be a triple-negative spindle cell carcinoma, underwent a modified radical mastectomy. After the first line chemotherapy with Cyclophosphamide and Epirubicin, multiple metastases in bilateral lung and left anterior thoracic wall appeared. After disease progressed with therapy of Bevacizumab combined with Albumin-bound Paclitaxel and Cisplatin, we treated the patient with Apatinib according to her VEGFR expression, which showed nearly complete response and controllable and tolerated side effects. Next-generation sequencing analysis of the tumor specimen and real time ctDNA was performed to observe the mutated gene numbers matched with therapeutic effect. The present case can help to provide a new and effective therapy strategy to treat advanced spindle cell carcinoma.

## INTRODUCTION

Metaplastic carcinoma is rare, accounting for less than 1% of primary tumors of the breast. Because the various form of differentiation along a multitude of cell lines, it is a frequent source of diagnostic difficulty. Patients tend to present with higher tumor size and stage than conventional invasive mammary carcinoma. Large lesions can be tethered to the skin and/or chest wall. Spindle cell carcinoma is characterized by spindle-shaped cells arranged in a variety of architectural patterns, and is a rare subtype of metaplastic carcinoma, accounting for about 0.024%-0.5%of all breast cancer [1–4]. Surgery and radiotherapy may be effective in the local control of low-grade spindle cell carcinoma. However, there are approximately up to 46% of these patients whose disease will recur at distant sites [5]. Effective chemotherapy specific for metaplastic carcinoma does not exist until now. New therapy is urgently required.

Recently, it has been demonstrated vascular endothelial growth factor (VEGF) and its receptor (VEGFR) are involved in angiogenesis of sarcomas and play a crucial role in tumor progression. Thus, angiogenesis is one of the potential targets, which is already an accepted therapy target of more prevalent cancers. A growing number of tyrosine kinase inhibitors (TKIs) towards VEGFR are being applied and many of them have therapeutic effect in different types of malignancies [6, 7]. Apatinib, a novel small molecule tyrosine kinase inhibitor, selectively inhibits VEGFR-2 and also moderately inhibits c-Kit and c-Src tyrosine kinase. By binding to VEGFR-2, Apatinib inhibits the effects of VEGF binding and subsequent VEGFR-2 auto-phosphorylation. In addition, Apatinib-mediated VEGFR-2 inhibition also appears to inhibit downstream phosphorylated extracellular signal-regulated kinase. Through this inhibition, Apatinib plays antiangiogenic and antitumor roles [8]. Apatinib has been recommended as third-line treatment for metastatic gastric cancer patients, showed improved progression-free survival (PFS) and overall survival (OS) in pretreated patients with metastatic gastric cancer who had experienced treatment failure with two or more chemotherapy regimens [9]. Moreover, the drug is currently being tested in patients with breast or lung cancers [10]. In this study, we aim to present our initial clinical experience with Apatinib for an advanced spindle cell carcinoma patient who experienced chemotherapy failure and describe its potential benefits and toxicity profiles (Hematological and non-hematological).

## CASE REPORT

**Figure 1:**
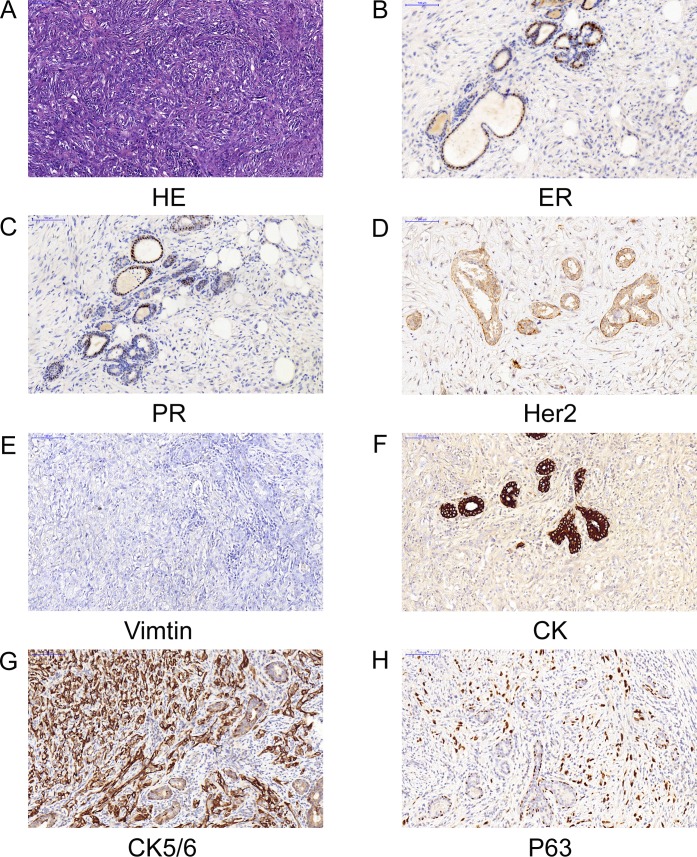
Pathology of biopsy and surgical resections indicated a triple-negative spindle cell carcinoma **A.** Hematoxylin and eosin (HE) of biopsy.**B.**.**C.D.E.F.G.H.**) Immunohistochemisty of surgical resections. ER (−);PR(−);Her2(−);Vimtin(−); CK(−);CK5/6(+);P63(+),(magnification 200×).

**Figure 2:**
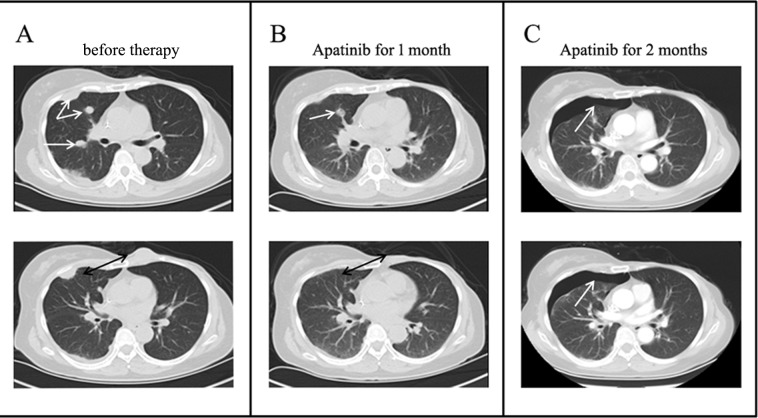
Chest CT scans before and after Apatinib therapy **A.** CT scans at different layers before Apatinib therapy revealed multiple metastases in right lung, pleura and chest wall; **B.** After 1 cycle Apatinib treatment, CT scans showed that the metastatic nodules became smaller or almost disappeared; **C.** After another therapy cycle, pulmonary metastasis further decreased and pneumatothorax appeared at the same site of pleura metastasis.

**Figure 3:**
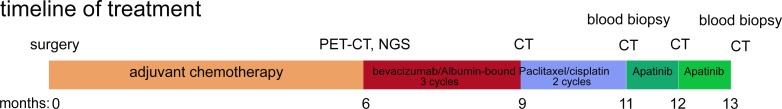
the various treatments the patient received when recurrence for multiple metastases in bilateral lung and left anterior thoracic wall as well as the duration of each treatment

This patient is a 52- year- old woman, who had experienced a benign tumorectomy of left mammary glands 4 years ago. One and a half years ago, she found a lump sized like a peanut kernel in the left breast by chance, which enlarged gradually during the following one month to a lump sized like a common quail egg. Histopathology examination of biopsy sample revealed spindle cell carcinoma by H&E staining (Figure 1A). Subsequently, a modified radical mastectomy had been performed. Histopathology combined with immunohistochemisty results showed a triple-negative (ER/PR/HER2) spindle cell carcinoma (Figure 1). This tumor has a basal epithelial phenotype with expression of high molecular weight cytokeratins 5/6, and epithelial marker p63 are also expressed. The spindle-shaped cells are negative for both vimentin and cytokeratin. (Figure 1). These biomarkers also excluded a diagnosis of others spindle cell lesions such as sarcoma. After 6cycles of adjuvant chemotherapy with Cyclophosphamide and Epirubicin, a Positron Emission Tomography/computed tomography (PET-CT) scan was done. It indicated multiple metastases in bilateral lung and left anterior thoracic wall. For obtaining better treatment, she came to our hospital. Physical examination showed consciousness was clear, with poor spirit and chronic faces, no stained yellow on skin and mucous membrane, and no touched swelling of superficial lymph nodes. The patient had got Asthma in 1995 and had no history of hypertension, diabetes, and heart disease. Combining with her tumor history, performance and chemotherapy regimen, we first performed precise diagnosis with genetic testing. The mRNA expression levels of several angiogenesis related genes and classic mutation sites of EGFR were tested by liquid phase chip and ARMS PCR using her surgery sample, which showed a high expression level of Vascular Endothelial Growth Factor Receptor1 (VEGFR1) and a medium expression level of VEGFR2. Moreover, there is no classic mutation of EGFR (Table 1). We then carried out next generation sequencing (NGS) using the same surgery sample, which showed numbers of mutated genes but no matched targeted therapy (Table 2). Aiming at the high VEGFR expression, bevacizumab combined with chemotherapy of Albumin-bound Paclitaxel and Cisplatin was started one week later. Metastatic nodules in lungs became smaller revealed by CT scanning after 3 cycles. However, after another 2cycles, CT scan showed disease progression at all metastatic sites with increased diameters and pleural effusion. Finally, because of the medium expression of VEGFR2, the patient was treated with Apatinib after signing informed consent and confirmed by the hospital ethics committee approval. Before Apatinib therapy, metastatic nodules in the middle lobe of right lung, pleura and left anterior thoracic wall were found by CT scan (Figure 2A). Remarkably, the mass in the left anterior thoracic wall shrank significantly only one week after 500mg/day Apatinib administration. After 1 cycle Apatinib treatment, CT scan showed that the metastatic nodules in pleura, left anterior thoracic wall and bilateral lungs became smaller or almost disappeared. In addition, pleural effusion was improved significantly (Figure 2B). Following an additional one month of Apatinib treatment, pulmonary metastasis further decreased and so as that in pleura and left anterior thoracic wall. It's worth noting that pneumatothorax appeared because of shrinkage of the pleura lesion, which could be controlled by symptomatic treatment (Figure 2C). To monitor the real-time effect of Apatinib on patient's gene mutation status, we carried out NGS detection of circulating tumor DNA (ctDNA) from peripheral blood before therapy and after 2 months therapy. The two NGS results were consistent with therapeutic effect, in which number of mutated genes decreased after Apatinib therapy (Table 3). During the therapy, the drug dose was decreased to 250mg/day due to hypertension and hand-foot syndrome until they were totally tolerated. Until now, therapy with Apatinib was continued for 8 months, and the patient was in good general condition after the initiation of Apatinib (data lock, January 2016). The whole timeline of treatment is organized as Figure 3.

**Table 1:** Test of mRNA expression and mutation status of known target

item	data	result	method
**HER2/NEU**	≥1.9%	low	liquid phase chip
**VEGFR1**	≥78.8%	high	liquid phase chip
**VEGFR2**	≥59.6%	medium	liquid phase chip
**VEGFR3**	≥32.9%	low	liquid phase chip
**EGFR exon18**	Wild type	No mutation	ARMS PCR
**EGFR exon19**	Wild type	No mutation	ARMS PCR
**EGFR exon20**	Wild type	No mutation	ARMS PCR
**EGFR exon21**	Wild type	No mutation	ARMS PCR

*Note:*HER2/NEU: Human Epidermal growth factor Receptor-2; VEGFR: Vascular Endothelial Growth Factor Receptor; EGFR: Epidermal Growth Factor Receptor

**Table 2:** NGS results of surgery sample

gene	base	amino acid	frequency
**MUTYH**	c.[1373T>C]	p.[L458P]	3.03%
**MED12**	c.[3236C>T]	p.[A1079V]	3.12%
**HSD17B3**	c.[383T>C]	p.[L128S]	3.79%
**CDK8**	c.[515-2A>G]	-	3.42%
**KMT2D**	c.[4474C>T]	p.[Q1492*]	3.14%
**AR**	c.[164T>A]	p.[L55Q]	3.86%
**ATRX**	c.[3472G>A]	p.[A1158T]	5.84%

*Note:* NGS: Next Generation Sequencing; MUTYH: mutY DNA glycosylase; MED12: mediator complex subunit 12; HSD17B3: hydroxysteroid (17-beta) dehydrogenase 3; CDK8: cyclin-dependent kinase 8; KMT2D: lysine methyltransferase 2D; AR: androgen receptor; ATRX: alpha thalassemia/mental retardation syndrome X-linked

**Table 3:** NGS results of ctDNA before and after Apatinib therapy

	Before therapy		
gene	base	amino acid	frequency
**RPL22**	c.[37delA]	p.[K13fs*5]	2.27%
**FLT4**	c.[2632G>A]	p.[V878M]	1.02%
**GNAQ**	c.[286A>T]	p.[T96S]	3.09%
**SYK**	c.[1091A>G]	p.[K364R]	1.08%
**FPGS**	c.[1307C>T]	p.[A436V]	1.21%
**KMT2D**	c.[11714A>T]	p.[Q3905L]	1.07%
**Apatinib 2 months therapy**			
**gene**	base	amino acid	frequency
**CCND3**	c.[48C>T]	p.[R50W]	1.11%
**MSH3**	c.[178_179ins CCGCAGCGC]	p.[Q3905L]	45.6%

*Note:* NGS: Next Generation Sequencing; RPL22: ribosomal protein L22; FLT4: fms related tyrosine kinase 4; GNAQ: G protein subunit alpha q; SYK: spleen tyrosine kinase; FPGS: folylpolyglutamate synthase; KMT2D: lysine methyltransferase 2D; CCND3: cyclin D3; MSH3: mutS homolog 3.

## DISCUSSION

Therapy of advanced spindle cell carcinoma has not developed until now except those classic chemotherapy regimens. There are some failed cases about spindle cell carcinoma. Either Bevacizumab or paclitaxel therapy could control disease progression [10, 11]. Recently, there were reports that EGFRs are over-expressed in spindle cell carcinomas, which indicated that TKIs are potential for spindle cell carcinoma therapy [12,13]. We had tested several angiogenesis related genes and classic mutation sites of EGFR for this patient and there is no mutation at any site of EGFR but VEGFR1 and VEGFR2 are over-expressed (Table 1). Expression of VEGFRs in the spindle cell carcinoma has not been fully explored nor the targeted therapy. A study about esophageal spindle cell carcinoma showed that VEGFR1 and VEGFR2 were upregulated in an established cell line from the metastasis esophageal spindle cell carcinoma and VEGF attenuated effect of Cisplatin [14].

As a novel and potent tyrosine kinase inhibitor, Apatinib selectively binds to and inhibits VEGFR-2. It could effectively inhibit VEGF-stimulated endothelial cell migration as well as proliferation and decrease tumor micro vessel density [15].In addition, Apatinib could prevent multidrug resistance conferred by ABCB1 and ABCG2 proteins [16]. Up to now, Apatinib has been successfully applied in advanced gastric cancer as a third-line treatment. Results from a recent published phase II randomized trial demonstrated that both OS and PFS were prolonged in heavily pretreated gastric cancer patients taking Apatinib 850 mg once daily than those taking placebo (median OS 4.8*vs*. 2.5 months, and median PFS 3.7 *vs*. 1.4 months) [8]. As to side effect, it was overall well tolerable for patients to take and only a limited number of patients had a partial response including hand-foot syndrome and hypertension. Severe hematologic toxicities were occasional. Research showed that the average survival benefit of Apatinib to pretreated Chinese gastric cancer patients (1.8 months) appears to be in the same range of that of Ramucirumab in the Western population (1.6 months) [9]. Other than for gastric cancer, Apatinib's applications for breast and non small cell lung cancer have been currently tested. Single-agent of Apatinib exhibited objective efficacy in heavily pretreated, metastatic non-triple-negative breast cancer with manageable toxicity [17, 18].

To our best knowledge, this is the first report of successfully using Apatinib to treat advanced spindle cell carcinoma of breast. Efficacy evaluation of the patient was nearly complete response (CR), which indicated that Apatinib is more effective than bevacizumab for this patient. Both as anti-angiogenic agent, Apatinib and bevacizumab work through different mechanisms. Bevacizumab is a recombinant, humanized monoclonal antibody that binds to VEGF-A, thus inhibiting angiogenesis. Bevacizumab is effective mainly in combination with chemotherapeutic drugs and potentiates chemotherapy not by inhibiting angiogenesis but abrogates the reactive resistance mediated by VEGF and HIF-1 and sensitizes both endothelial and cancer cells to therapy [19]. While Apatinib is a tyrosine kinase inhibitor targeted VEGFR2, which abrogates the interaction of VEGF with VEGFR and directly inhibits angiogenesis. Moreover, side effects of Apatinib were controllable and well tolerated. All results implied that Apatinib might be a safe and effective oral targeted drug on patients with advanced spindle cell carcinoma of breast, especially for those experienced failure of chemotherapy or with poor physical condition. In order to explore the potential associated gene mutation, we performed NGS before and after Apatinib therapy. The profile of gene mutations varies between the breast cancer specimen and ctDNA because of tumor's heterogeneity. In addition, ctDNA comes from degradation of cancer cell DNA and it would change during different stages of tumor status. Moreover, chemotherapy and targeted therapy drugs could impact the profile of gene mutation. We monitored the profile of gene mutation of this patient using NGS in order to explore the potential gene mutation impacted by Apatinib, and obviously we didn't find one from this patient and so that we need additional observation and research. To further investigate the role of Apatinib in advanced spindle cell carcinoma, large sample and additional clinical trials are needed.

## MATERIALS AND METHODS

### Histopathology examination

Histopathology examination of biopsy samples were cut into slices, fixed immediately in formalin (10%) and then embedded in paraffin. Serial 5μm thick sections were cut from paraffin blocks and attached to slides. Immunohistochemical studies were carried out with Leica BOND-MAX™, an IHC automatic staining system, according to the manufacturer's instruction.

### NGS-based assay

Tumor DNA was extracted from FFPE samples using QIAamp DNA FFPE Tissue Kit and Qiagen's DNEasy Blood and Tissue Extraction Kit (Qiagen), respectively, according to the manufacturer's instructions. Genomic DNA from peripheral blood was purified using QIAamp DNA Blood Mini Kit (Qiagen). All FFPE tissue samples were reviewed by a qualified pathologist to ensure > 70% tumor content. DNA purity and concentration were determined by the NanoDrop2000 spectrophotometer and Qubit 2.0 Fluorometer with Quant-IT dsDNA HS Assay Kit (Thermo Fisher Scientific), respectively. The quality of genomic DNA from tumor tissue and peripheral blood was assessed by agarose gel electrophoresis and the size distribution of circulating DNA was evaluated on a 2100 Bioanalyzer using the DNA 1000 Kit (Agilent).

Library construction with tumor tissue DNA and peripheral blood DNA was performed using 1μg of DNA sheared by an ultrasonoscope to generate fragments with a peak of 250 bps, followed by end repair, a tailing and ligation to the Illumina-indexed adapters according to the standard library construction protocol. Target enrichment was performed on a custom sequence capture-probe (Nimblegen, USA) which targeted 7,708 exons of 508 cancer-related genes and 78 introns from 19 genes recurrently rearranged in solid tumor representing ~1.7Mb of the human genome in total Supplementary Table S1. Sequencing was performed with 2×101 bp paired-end reads and 8-bp index read on an Illumina Hiseq 2500 platform (Illumina, San Diego, USA) using the manufacturer's protocols.

Primary sequence data were first processed by filtering adaptor sequences and removing low-quality reads using the SOAPnuke software (http://soap.genomics.org.cn/) developed by BGI, and aligned to build hg19 of the NCBI reference genome assembly using BWA aligner v0.6.2-r126. PCR duplicate reads were removed by PICARD v1.98. Local realignment and base quality score recalibration were performed using GATK v2.3-9 and poorly mapped reads were removed based on the recalibration result. SNVs were detected by Mutect and SOMATK-SNV (developed by BGI, manuscript in preparation) and Indel (small insertions and deletions) were detected by GATK and SOMATK-INDEL (developed by BGI, manuscript in preparation). CNV calling was done by CONTRA v2.0.4.

## SUPPLEMENTARY MATERIALS TABLES



## Abbreviations

ER: Estrogen Receptor
PR: Progesterone Receptor
Her2: Human Epidermal growth factor Receptor 2
VEGFR: Vascular Endothelial Growth Factor Receptor
NGS: Next Generation Sequencing
TKIs: tyrosine kinase inhibitors

## References

[1] Cheah Alison L, Billings Steven D, Rowe J. Jordi (2016). Mesenchymal tumours of the breast and their mimics: a review with approach to diagnosis. Pathology.

[2] Khan HN1, Wyld L, Dunne B, Lee AH, Pinder SE, Evans AJ, Robertson JF (2003). Spindle cell carcinoma of the breast : a case series of a rare histological subtype. Eur J Surg Oncol.

[3] Okushiba S, Minagawa H, Shimizu M, Ambo Y, Kaji M, Omi M, Itoh K, Kondo S, Katoh H (2001). A case of spindle cell carcinoma of the breast—long survival achieved by multiple surgical treatment. Breast Cancer.

[4] Kitada M, Hayashi S, Matsuda Y, Ishibashi K, Oikawa K, Miyokawa N (2014). Spindle cell carcinoma of the breast as complex cystic leision:a case report. Cancer Biol Me.

[5] Carter MR, Hornick JL, Lester S, Fletcher CD (2006). Spindle cell (sarcomatoid) carcinoma of the breast.A clinicopathologie and immunohistochem—teal analysis of 29 cases. Am J Surg Pathol.

[6] Sleijfer S, Ray-Coquard I, Papai Z, Le Cesne A, Scurr M, Schöffski P, Collin F, Pandite L, Marreaud S, De Brauwer A, van Glabbeke M, Verweij J, Blay JY (2009). Pazopanib, a multikinase angiogenesis inhibitor, in patients with relapsed or refractory advanced soft tissue sarcoma: a phase II study from the European organization for research and treatment of cancer-soft tissue and bone sarcoma group (EORTC study 62043). J Clin Oncol.

[7] Lammli J, Fan M, Rosenthal HG, Patni M, Rinehart E, Vergara G, Ablah E, Wooley PH, Lucas G, Yang SY (2012). Expression of vascular endothelial growth factor correlates with the advance of clinical osteosarcoma. Int Orthop.

[8] Haijun Zhang (2015). Apatinib for molecular targeted therapy in tumor. Drug Des Devel Ther.

[9] Li J, Qin S, Xu J, Guo W, Xiong J, Bai Y, Sun G, Yang Y, Wang L, Xu N, Cheng Y, Wang Z, Zheng L (2013). Apatinib for Chemotherapy-Refractory Advanced Metastatic Gastric Cancer: Results from a Randomized, Placebo-Controlled, Parallel-Arm, Phase II Trial. J Clin Oncol.

[10] Fontanella C, Ongaro E, Bolzonello S, Guardascione M, Fasola G, Aprile G (2014). Clinical advances in the development of novel VEGFR2 inhibitors. Ann Transl Med.

[11] Nakano M, Yamamoto Y, Kawasoe T, Hayashi M, Sueta A, Honda Y, Iyama K, Iwase H (2009). A case of spindle cell carcinoma of the breast including metaplastic lesion with poor prognosis. Gan To Kagaku Ryoho [Article in Japanese].

[12] Murata T, Fujii M, Aoki M, Oda K (2011). A case of spindle cell carcinoma of the breast, in which irinotecan was effective against respiratory failure due to pulmonary metastases. Gan To Kagaku Ryoho [Article in Japanese].

[13] Leibl S, Moinfar F (2005). Metaplastic breast carcinomas are negative for Her-2 but frequently express EGFR(Her-1):potential relevance to adjuvant treatment with EGFR tyrosine kinase inhibitors. J Clin Pathol.

[14] Gilbert JA, Goetz MP, Reynolds CA, Ingle JN, Giordano KF, Suman VJ, Blair HE, Jenkins RB, Lingle WL, Reinholz MM, Adjei AA, Ames MM (2008). Molecular analysis of metaplastic breast carcinoma:high EGFR copy number via aneusomy. Mol Cancer Ther.

[15] Nakatani H, Akimori T, Takezaki Y, Hanazaki K (2010). Vascular endothelial growth factors and their receptors in the novel human cell line, HN-Eso-1, established from esophageal spindle cell carcinoma. J Med Invest.

[16] Li J, Zhao X, Chen L, Guo H, Lv F, Jia K, Yv K, Wang F, Li C, Qian J, Zheng C, Zuo Y (2010). Safety and pharmacokinetics of novel selective vascular endothelial growth factor receptor-2 inhibitor YN968D1 in patients with advanced malignancies. BMC Cancer.

[17] Mi YJ, Liang YJ, Huang HB, Zhao HY, Wu CP, Wang F, Tao LY, Zhang CZ, Dai CL, Tiwari AK, Ma XX, To KK, Ambudkar SV (2010). Apatinib (YN968D1) reverses multidrug resistance by inhibiting the efflux function of multiple ATP-binding cassette transporters. Cancer Res.

[18] Hu X, Cao J, Hu W, Wu C, Pan Y, Cai L, Tong Z, Wang S, Li J, Wang Z, Wang B, Chen X, Yu H (2014). Multicenter phase II study of apatinib in non-triple-negative metastatic breast cancer. BMC Cancer.

[19] Blagosklonny MV (2005). How Avastin Potentiates Chemotherapeutic Drugs Action and Reaction in Antiangiogenic Therapy. Cancer Biol Ther.

