# Supplementary material for: Response to apatinib in chemotherapy-failed advanced spindle cell breast carcinoma
Source: Oncotarget. 2016 Oct 11;7(44):72373–9. doi: 10.18632/oncotarget.12568 (PMC5342168; doi:10.18632/oncotarget.12568)
Supplement: Supplementary file 1 [file oncotarget-07-72373-s001.pdf]

Response to apatinib in chemotherapy-failed advanced spindle cell breast carcinoma

Supplementary Material

Supplementary Table S1

Gene list for NGS assay in this study									
**ABL1**	**C1R**	**DIS3**	**FGF19**	**HSPA4**	**MIR142**	**PAX5**	**RB1**	**SRSF2**	
**ABL2**	**C1S**	**DNMT1**	**FGF23**	**IDH1**	**MITF**	**PBRM1**	**REL**	**SSTR2**	
**ACVR1B**	**CARD11**	**DNMT3A**	**FGF3**	**IDH2**	**MLH1**	**PCBP1**	**RET**	**STAG2**	
**ACVR2A**	**CASP8**	**DOT1L**	**FGF4**	**IFNAR1**	**MLH3**	**PCM1**	**RHEB**	**STAT4**	
**AJUBA**	**CBFB**	**DUSP6**	**FGF6**	**IFNAR2**	**MLL**	**PDGFRA**	**RICTOR**	**STAT5B**	
**AKT1**	**CBL**	**EDNRA**	**FGF7**	**IGF1**	**MLL2**	**PDGFRB**	**RNASEL**	**STK11**	
**AKT2**	**CBLB**	**EGFR**	**FGFR1**	**IGF1R**	**MLL3**	**PDK1**	**RNF43**	**SUFU**	
**AKT3**	**CBR1**	**EGR3**	**FGFR2**	**IGF2**	**MLL4**	**PHF6**	**ROBO1**	**SUZ12**	
**ALK**	**CCND1**	**EIF4A2**	**FGFR3**	**IKBKB**	**MPL**	**PIGF**	**ROBO2**	**SYK**	
**ALOX12B**	**CCND2**	**ELAC2**	**FGFR4**	**IKBKE**	**MRE11A**	**PIK3C2A**	**ROS1**	**TAF1**	
**ANGPT1**	**CCND3**	**ELF3**	**FH**	**IKZF1**	**MS4A1**	**PIK3C2B**	**RPA1**	**TBL1XR1**	
**ANGPT2**	**CCNE1**	**EML4**	**FLCN**	**IL7R**	**MSH2**	**PIK3C2G**	**RPL22**	**TBX3**	
**APC**	**CD79A**	**EP300**	**FLT1**	**INHBA**	**MSH3**	**PIK3C3**	**RPL5**	**TEK**	
**APCDD1**	**CD79B**	**EPCAM**	**FLT3**	**IRF4**	**MSH4**	**PIK3CA**	**RPS14**	**TERT**	
**AR**	**CDC25C**	**EPHA2**	**FLT4**	**IRS2**	**MSH5**	**PIK3CB**	**RPS6KB1**	**TET2**	
**ARAF**	**CDC42**	**EPHA3**	**FNTA**	**ITGB2**	**MSH6**	**PIK3CG**	**RPTOR**	**TFG**	
**ARFRP1**	**CDC73**	**EPHA5**	**FOXA1**	**JAK1**	**MSR1**	**PIK3R1**	**RUNX1**	**TGFBR2**	
**ARHGAP35**	**CDH1**	**EPHB1**	**FOXA2**	**JAK2**	**MTOR**	**PIK3R2**	**RUNX1T1**	**TIPARP**	
**ARID1A**	**CDK12**	**EPHB2**	**FOXL2**	**JAK3**	**MUC1**	**PLK1**	**RXRA**	**TLR4**	
**ARID1B**	**CDK2**	**EPHB6**	**FPGS**	**JUN**	**MUTYH**	**PML**	**RXRB**	**TMEM127**	
**ARID2**	**CDK4**	**EPPK1**	**FUBP1**	**KAT6A**	**MYC**	**PMS1**	**RXRG**	**TNFAIP3**	
**ARID5B**	**CDK6**	**ERBB2**	**FYN**	**KDM5A**	**MYCL1**	**PMS2**	**SDHAF2**	**TNFRSF14**	
**ASXL1**	**CDK8**	**ERBB3**	**GAB2**	**KDM5C**	**MYCN**	**PNRC1**	**SDHB**	**TNFRSF8**	
**ATM**	**CDKN1A**	**ERBB4**	**GATA1**	**KDM6A**	**MYD88**	**POLQ**	**SDHC**	**TNFSF11**	
**ATR**	**CDKN1B**	**ERCC2**	**GATA2**	**KDR**	**NAV3**	**PPP2R1A**	**SDHD**	**TNFSF13B**	
**ATRX**	**CDKN2A**	**ERCC3**	**GATA3**	**KEAP1**	**NBN**	**PRDM1**	**SEMA3A**	**TOP1**	
**AURKA**	**CDKN2B**	**ERG**	**GID4**	**KIF1B**	**NCOA1**	**PRKAA1**	**SEMA3E**	**TOP2A**	
**AURKB**	**CDKN2C**	**ESR1**	**GNA11**	**KIF5B**	**NCOA2**	**PRKAR1A**	**SETBP1**	**TOP2B**	
**AXIN1**	**CDX2**	**ETV1**	**GNA13**	**KIT**	**NCOR1**	**PRKCA**	**SETD2**	**TP53**	
**AXIN2**	**CEBPA**	**ETV6**	**GNAQ**	**KLF4**	**NEK11**	**PRKCB**	**SF1**	**TRAF7**	
**AXL**	**CFLAR**	**EWSR1**	**GNAS**	**KLHL6**	**NF1**	**PRKCG**	**SF3B1**	**TSC1**	
**B2M**	**CHD1**	**EXT1**	**GNRHR**	**KRAS**	**NF2**	**PRKDC**	**SH2B3**	**TSC2**	
**B4GALT3**	**CHD2**	**EXT2**	**GPR124**	**LCK**	**NFE2L2**	**PRSS8**	**SIN3A**	**TSHR**	

**BACH1**	**CHD4**	**EZH2**	**GRIN2A**	**LIMK1**	**NFE2L3**	**PSMB1**	**SLAMF7**	**TSHZ2**
**BAK1**	**CHEK1**	**FAM123B**	**GRM3**	**LRRK2**	**NFKBIA**	**PSMB2**	**SLC4A1**	**TSHZ3**
**BAP1**	**CHEK2**	**FAM46C**	**GSK3B**	**LYN**	**NKX2-1**	**PSMB5**	**SLIT2**	**TUBA1A**
**BARD1**	**CHUK**	**FANCA**	**H3F3A**	**MALAT1**	**NKX3-1**	**PTCH1**	**SMAD2**	**TUBB**
**BCL2**	**CIC**	**FANCC**	**H3F3C**	**MAP2K1**	**NOTCH1**	**PTCH2**	**SMAD3**	**TUBD1**
**BCL2A1**	**CRBN**	**FANCD2**	**HCK**	**MAP2K2**	**NOTCH2**	**PTEN**	**SMAD4**	**TUBE1**
**BCL2L1**	**CREBBP**	**FANCE**	**HDAC1**	**MAP2K4**	**NOTCH3**	**PTP4A3**	**SMARCA1**	**TUBG1**
**BCL2L11**	**CRIPAK**	**FANCF**	**HDAC2**	**MAP3K1**	**NOTCH4**	**PTPN11**	**SMARCA4**	**TYR**
**BCL2L2**	**CRKL**	**FANCG**	**HDAC3**	**MAP3K13**	**NPM1**	**PTPRD**	**SMARCB1**	**U2AF1**
**BCL6**	**CRLF2**	**FANCI**	**HDAC4**	**MAPK1**	**NR3C1**	**RAC1**	**SMARCD1**	**USP9X**
**BCOR**	**CROT**	**FANCL**	**HDAC6**	**MAPK3**	**NRAS**	**RAC2**	**SMC1A**	**VEGFA**
**BCORL1**	**CSF1R**	**FANCM**	**HDAC8**	**MAPK8**	**NSD1**	**RAD21**	**SMC3**	**VEGFB**
**BCR**	**CTCF**	**FAT3**	**HGF**	**MAPK8IP1**	**NTRK1**	**RAD50**	**SMO**	**VEZF1**
**BLM**	**CTLA4**	**FBXW7**	**HIF1A**	**MAX**	**NTRK2**	**RAD51**	**SOCS1**	**VHL**
**BMPR1A**	**CTNNA1**	**FCGR1A**	**HIST1H1C**	**MC1R**	**NTRK3**	**RAD51B**	**SOX10**	**WHSC1L1**
**BRAF**	**CTNNB1**	**FCGR2A**	**HIST1H2BD**	**MCL1**	**NUP93**	**RAD51C**	**SOX17**	**WISP3**
**BRCA1**	**CUL4A**	**FCGR2B**	**HIST1H3B**	**MDM2**	**PAK3**	**RAD51D**	**SOX2**	**WWP1**
**BRCA2**	**CUL4B**	**FCGR2C**	**HNF1A**	**MDM4**	**PAK7**	**RAD52**	**SOX9**	**XIAP**
**BRIP1**	**CYLD**	**FCGR3A**	**HRAS**	**MECOM**	**PALB2**	**RAD54L**	**SPEN**	**XPA**
**BTG1**	**CYP17A1**	**FCGR3B**	**HRH2**	**MED12**	**PARP1**	**RAF1**	**SPOP**	**XPC**
**BTK**	**DAXX**	**FGF10**	**HSD17B3**	**MEF2B**	**PARP2**	**RARA**	**SPRY4**	**XPO1**
**C11orf30**	**DDR1**	**FGF12**	**HSD3B2**	**MEN1**	**PARP3**	**RARB**	**SRC**	**XRCC3**
**C1QA**	**DDR2**	**FGF14**	**HSP90AA1**	**MET**	**PARP4**	**RARG**	**SRD5A2**	**YES1**
**ZNF217**	**ZNF703**	**ZRSR2**	**WT1**					
